# Global, regional, and national burden of endometriosis among women of reproductive age, 1990–2021: Insights from the global burden of disease study 2021

**DOI:** 10.1371/journal.pone.0337074

**Published:** 2025-11-26

**Authors:** Jun Zhang, Mengduan Pang, Ling Li, Chuanjia Guo

**Affiliations:** 1 Department of obstetrics and gynecology, first affiliated hospital of dalian medical university, dalian, china; 2 Department of rheumatology and immunology, first affiliated hospital of dalian medical university, dalian, china; Athens Medical Group, Psychiko Clinic, GREECE

## Abstract

**Background:**

Endometriosis is a common gynecological disorder among women of reproductive age worldwide. This study aims to examine global patterns of endometriosis disease burden among reproductive-aged women and to evaluate its correlation with socioeconomic development indices.

**Methods:**

This study conducted a comprehensive analysis of endometriosis disease burden using epidemiological parameters from the Global Burden of Disease (GBD) 2021 database, including incidence rates, prevalence rates, Disability-Adjusted Life Years (DALYs), and corresponding age-standardized rates. Through stratified analyses at global, regional, and national levels, we systematically evaluated the disease burden patterns among reproductive-aged women and performed correlation analysis with socioeconomic development indices.

**Results:**

We found that the highest incidence of endometriosis among women of reproductive age globally occurs in the 20–24 age group, with an incidence rate of 399.49 per 100,000 in 1990 and 304.31 per 100,000 in 2021. The results show that the global disease burden of endometriosis is mainly influenced by population size, followed by epidemiological changes. Compared to countries with a medium Social Development Index (SDI), the disease burden of endometriosis in low and high SDI regions fluctuated significantly from 1990 to 2021. In most countries with SDI values between 0.2 and 0.6, the burden of endometriosis showed a gradual decline.

**Conclusion:**

Endometriosis remains a significant public health issue for women of reproductive age globally. Although the global disease burden of endometriosis among women of reproductive age showed a slight decline from 1990 to 2021, the disease burden continues to fluctuate in certain regions and countries. In light of the differences in the disease burden of endometriosis across regions and countries, regionalized disease management strategies are expected to be developed in the future.

## Introduction

Endometriosis is an estrogen-dependent chronic inflammatory disease characterized by the migration of endometrial tissue. This can affect the endocrine system, reproductive system, nervous system, and other systems, leading to a range of clinical symptoms. It is considered a complex disease that involves multiple systems [1]. Endometriosis, as a common benign gynecological disease, is often overlooked. Its main clinical manifestations include menstrual pain, dyspareunia, and lower abdominal pain [2]. Furthermore, endometriosis also affects female fertility and is one of the leading causes of female infertility [3]. Endometriosis is often accompanied by inflammatory changes in the pelvic cavity. Multiple pro-inflammatory cytokines can impair sperm motility and capacitation, and oxidative stress can disrupt the acrosome reaction, thereby hindering sperm–oocyte interaction and fertilization [4]. According to reports, endometriosis affects approximately 10% of women worldwide, with a prevalence rate as high as 50% among individuals affected by infertility [5]. Endometriosis not only jeopardizes reproductive health and quality of life at the individual level but also has a considerable impact on population health. Individually, its typical phenotypes—chronic pelvic pain, dysmenorrhea, and dyspareunia—often coexist with impaired fertility, together with psychological comorbidities such as anxiety, depression and sleep disturbances, all of which adversely affect patients’ quality of life. At the population level, the constellation of high prevalence, diagnostic delay, and a tendency toward recurrence/chronicity cumulatively increases DALYs, constituting a persistent public health burden. Guided by health equity, narrowing disparities in disease burden among populations at different levels of socioeconomic development is essential to substantively reduce the overall societal burden of endometriosis. Overall, endometriosis has a significant negative impact on individuals, families, and public health, and imposes a substantial socioeconomic burden.

Women of reproductive age constitute the primary population affected by endometriosis, which is estimated to affect approximately 4–10% of women in this age group according to previous studies [6]. At present, the diagnosis of endometriosis remains a significant clinical challenge. Studies have shown that the average delay from the first onset of symptoms to a confirmed diagnosis is approximately 6.6 years [7]. The disease typically presents with pain-related symptoms, such as dysmenorrhea, dyspareunia, dyschezia, dysuria, and chronic pelvic pain. However, disparities in healthcare resources, diagnostic capacity, and awareness of endometriosis across regions mean that both clinicians and patients in some settings may fail to recognize these symptoms or associate them with endometriosis, indirectly contributing to the widespread problem of diagnostic delay. Furthermore, confirmation of the diagnosis often relies on invasive procedures such as laparoscopy, which further limits opportunities for early detection and intervention.

The effects of endometriosis are not limited to the reproductive system; it also negatively affects mental health, psychological well-being, and quality of life [8–11]. Additionally, the risk of cancer in these patients is also higher [12]. Recent studies suggest that the inflammatory processes associated with endometriosis may lead to endothelial dysfunction, and the incidence of cardiovascular diseases is also higher in these patients [13]. In women of reproductive age, endometriosis and infertility often occur together. It is reported that approximately 30% of women with endometriosis are also infertile. Compared to the unaffected population, the risk of infertility is up to four times higher in women with endometriosis [14]. Moreover, studies have shown that as endometriosis progresses, the success rates of various assisted reproductive treatments, including in vitro fertilization, also decrease [15].

A growing body of evidence indicates that the incidence of endometriosis exhibits epidemiological variability across different countries and regions. This variability is associated with multiple factors, such as genetic predisposition, the use of hormonal contraceptives, and occurrences of preterm birth [16,17]. Therefore, exploring the differences in the disease burden of endometriosis across countries and regions is of great significance. The updates to the GBD database not only provide rich data resources for epidemiological research but also highlight health disparities between different countries and regions. Previous studies have analyzed the disease burden of endometriosis using the GBD 2017 and GBD 2019 databases [18,19]. This study aims to clearly quantify the global burden of endometriosis among women of reproductive age and to describe its temporal trends and geographic heterogeneity. Furthermore, we seek to examine how the burden of endometriosis varies across countries with different levels of socioeconomic development. To achieve these goals, we utilized the most recent Global Burden of Disease 2021 database to assess trends over time and cross-country patterns, and to explore associations between disease burden indicators and socioeconomic metrics.

## Methods

### Study population and data sources

Reproductive-age women are those who have the ability to conceive. The reproductive-age female population is an important focus of research in the field of public health. Focusing on the latest health status and disease burden of reproductive-age women is essential, as it plays an important role in revising health policies, reducing maternal mortality, and improving reproductive healthy [20]. The World Health Organization defines the age range for reproductive-age women as 15–49 years. Endometriosis is a significant health issue for women of reproductive age, impacting both their physiological health and quality of life. Therefore, this study focuses on the disease burden of endometriosis in reproductive-age women.

GBD 2021 is a large-scale international collaborative research project organized by the World Health Organization, the Institute for Health Metrics and Evaluation at the University of Washington, and other institutions. GBD 2021 provides the latest epidemiological data on the burden of 371 diseases and injuries, offering detailed disease burden data at the global, national, and regional levels. Similar to previous research methods, data was collected through the GBD Results Tool (https://vizhub.healthdata.org/gbdresults/) and various epidemiological indicators of endometriosis in women aged 15–49 were extracted from the GBD 2021 database, including incidence, prevalence, and DALYs [21]. DALYs is a composite metric widely used to quantify the overall burden of disease. It integrates years of life lost (YLL) due to premature mortality with years lived with disability (YLD), thereby providing a comprehensive measure of the total healthy life years lost within a population as a result of a specific disease or injury. Elevated DALYs values indicate a substantial loss of healthy life expectancy in the affected population, signifying that such conditions are typically prioritized targets for public health intervention and policy development.

This study followed the definition and inclusion criteria for endometriosis described in the GBD 2021 methodology for gynecological non-fatal conditions. The case definition was based on the criteria of the ACOG guidelines: pelvic examination combined with confirmation by laparoscopy or laparotomy. Disease coding was mapped to the ICD-10 codes N80–N80.9 (including endometriosis and its site-specific classifications) used in GBD data processing. We included annual location–year observations for females aged 15–49 years from 1990 to 2021 and extracted incidence, prevalence, and DALYs rate measures from the GBD database.

To explore the differences in the disease burden of endometriosis across different age groups of reproductive-age women, we collected epidemiological data in five-year age intervals. Additionally, we gathered data from 21 GBD regions and 204 countries, along with their corresponding SDI. SDI as a composite index, incorporates multiple dimensions such as income, education, and demographics, providing a more comprehensive reflection of a country’s or region’s level of development. The calculation of SDI is based on three core indicators: income level, education level, and fertility rate. After standardization, an index value between 0 and 1 is calculated, with higher values indicating a higher level of social and demographic development. In GBD 2021, the 204 countries are divided into five SDI levels (high, high-middle, middle, low-middle, and low) based on their SDI values.

As this research is a secondary analysis based on the publicly available GBD 2021 database, ethical review was not necessary.

### Decomposition analysis

To reveal the key driving factors influencing the changes in the disease burden of endometriosis, we conducted a decomposition analysis based on the incidence, prevalence, and DALYs rates of reproductive-age women from 1990 to 2021. Specifically, we explored the impact of population size, epidemiological changes, and demographic structure changes on the variation in the disease burden of endometriosis. We employed the decomposition analysis method proposed by Das Gupta, which uses mathematical techniques to break down the overall change into independent and interactive effects of multiple factors, aiming to investigate the contribution of each factor [22].

Das Gupta decomposition analysis is a method that partitions the difference in overall rates into the contributions of multiple factors by symmetrically allocating interaction effects. This approach ensures that the decomposition results are not affected by the order of factors and can handle any number of influencing variables. Specifically, the Das Gupta decomposition breaks the observed overall rate difference into several intuitive component effects, with their relative contributions summing to 100%. The method assigns weights to each possible path of factor changes and, when calculating the importance of a particular factor, progressively introduces changes in other factors, which leads to a gradual reduction in that factor’s marginal contribution under counterfactual scenarios. Owing to its flexible framework and the intuitive visualization of results, it can be widely applied to the analysis of sociodemographic characteristics and differences in health outcomes [23,24].

### Frontier analysis

To assess the disease control situation of endometriosis in different countries and regions, we conducted a frontier analysis to compare the gap between the real-world and the achievable minimum disease burden. Frontier analysis is a best-value–based comparative method that identifies the lowest attainable burden of endometriosis at a given level of the SDI and quantifies the gap between observed performance and this benchmark. This approach effectively removes the confounding effects between development level and disease burden. In practice, it involves plotting the relationship between SDI and the DALYs in a scatterplot and overlaying a LOESS-smoothed frontier curve, thereby highlighting countries that continue to have a disproportionately high burden despite having the same SDI. First, we analyzed the non-linear relationship between the age-standardized DALYs from 1990 to 2021 in different countries and regions and their corresponding SDI indices, and then plotted a frontier curve. Next, we applied the Bootstrap method to perform 100 resampling iterations to generate different bootstrap samples, and fitted the data using LOESS. Finally, we calculated the effective difference between the actual disease burden at a given SDI value and the optimal frontier. This method can identify the potential areas for improvement in disease management across countries and regions.

### Statistical analysis

All data were processed, analyzed, and visualized using R software (version 4.4.1). P-value < 0.05 was considered statistically significant. And all visualizations were produced with the ggplot2 package.

## Results

### Changes in the endometriosis burden in 5 years of age between 1990 and 2021

**Fig 1:**
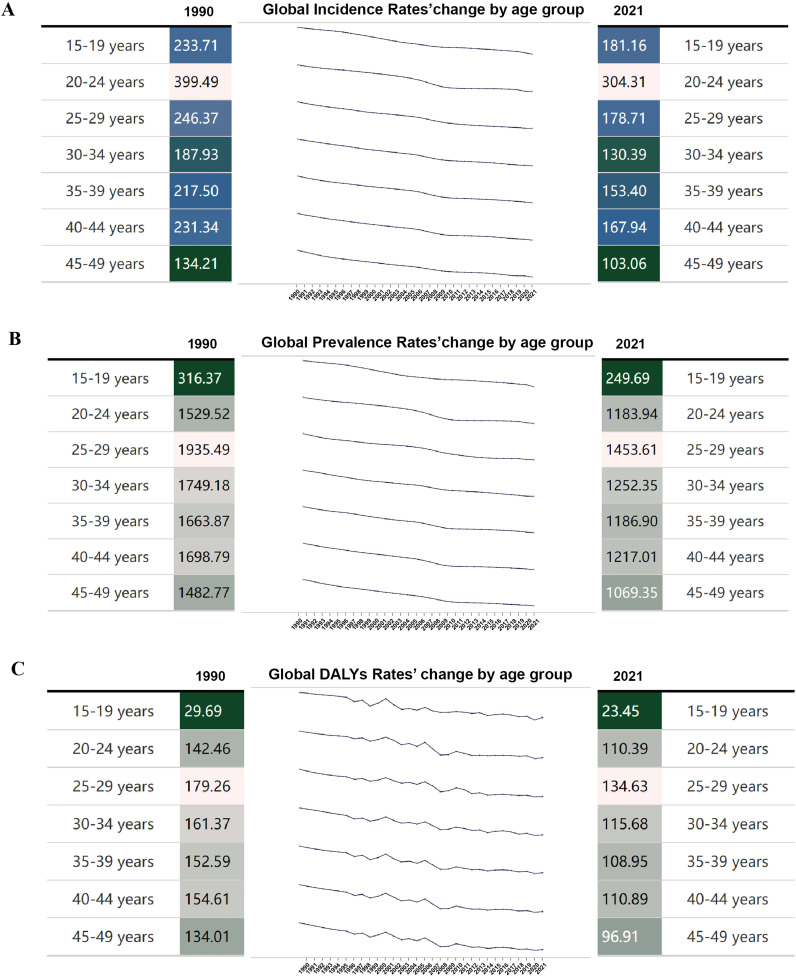
Global difference in disease burden of endometriosis at 5 years in women of reproductive age from 1990 to 2021. **(A)** Global endometriosis Incidence Rates(per 100,000 population) by age group. **(B)** Global endometriosis Prevalence Rates(per 100,000 population) by age group. **(C)** Global endometriosis DALYs Rates(per 100,000 population) by age group.

Fig 1 shows the trends in the incidence rate, prevalence rate, and DALYs rate of endometriosis among reproductive-age women, divided into 5-year age intervals, from 1990 to 2021. The results show that the incidence and prevalence of endometriosis among global reproductive-age women have generally shown a declining trend. It is worth noting that the 20–24 age group has the highest incidence of endometriosis, with an incidence rate of 399.49/100,000 in 1990 and 304.31/100,000 in 2021. Additionally, the incidence of endometriosis reached a second peak in the 40–44 age group, with an incidence rate of 231.34 per 100,000 in 1990 and 167.94 per 100,000 in 2021. The age group with the highest prevalence of endometriosis among global reproductive-age women was the 25–29 age group, with a prevalence of 1,935.49 per 100,000 in 1990 and 1,453.61 per 100,000 in 2021. The DALYs rate for endometriosis among global reproductive-age women fluctuated between 1990 and 2021 but, overall, gradually decreased.

### Decomposition Analysis

**Fig 2:**
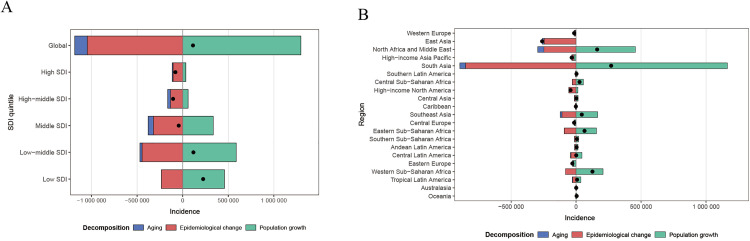
The decomposition analysis of endometriosis Incidence rates globally, across 5 SDI regions, and 21 regions from 1990 to 2021; The black dots represent the overall change values due to population growth, aging, and epidemiological changes. **(A)** Incidence rates globally and in 5 SDI regions; **(B)** Incidence rates in 21 GBD regions.

Fig 2 shows the results of the decomposition analysis conducted for the global population, 21 GBD regions, and 5 SDI regions, identifying the key factors contributing to differences in the burden of endometriosis. The result shows that the global endometriosis burden is mainly influenced by population size, followed by epidemiological changes. Compared to high and high-middle SDI regions, the burden of endometriosis in middle, low-middle, and low SDI regions are more heavily influenced by population size. Among the 5 SDI regions, the middle SDI region is most affected by aging, while the low SDI region is least affected by aging. In the 21 GBD regions, regions such as South Asia, North Africa and the Middle East, and Western Sub-Saharan Africa are more significantly influenced by population factors in terms of incidence rates. Meanwhile, South Asia, North Africa, and East Asia have a higher impact from epidemiological changes on incidence rates. The changes in endometriosis prevalence rates and DALYs rates are similar to the incidence rates result (S1 Fig).

### Frontier analysis

**Fig 3:**
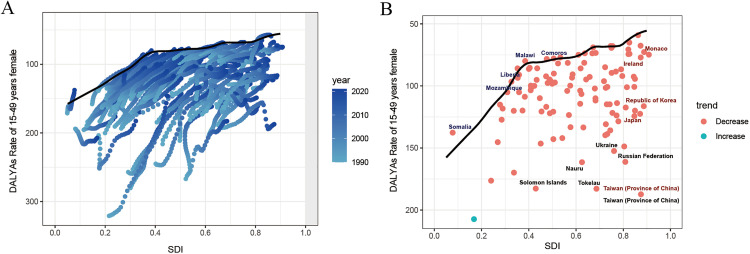
Frontier analysis of age-standardized DALYs rates for endometriosis. **(A)** Frontier analysis based on the SDI index and endometriosis DALYs rates from 1990 to 2021 in 204 countries; **(B)** Frontier analysis based on the SDI index and endometriosis DALYs rates in 204 countries in 2021. The black line represents the optimal endometriosis control situation for the SDI index.

Fig 3 illustrates the results of the frontier analysis for different countries, with the DALYs rate of the best-performing country set as the benchmark. Blue dots depict the trends in the burden of endometriosis for countries across different SDI levels from 1990 to 2021. The results show that compared to medium SDI countries, the disease burden fluctuations of endometriosis in low and high SDI regions were more significant between 1990 and 2021. In most countries with SDI value between 0.2 and 0.6, the disease burden of endometriosis showed a trend of annual improvement, with the ‘effective difference’ between them and the boundary gradually decreasing (Fig 3A). The disease burden of endometriosis in most countries showed a downward trend. Several medium SDI countries, including Comoros, Malawi, Liberia, and Mozambique, have DALYs rates closer to the boundary, indicating good disease control in these countries. However, in some countries, such as the Solomon Islands, Nauru, and Tokelau, the ‘effective difference’ between their DALYs rates and the boundary is larger (Fig 3B).

**Fig 4:**
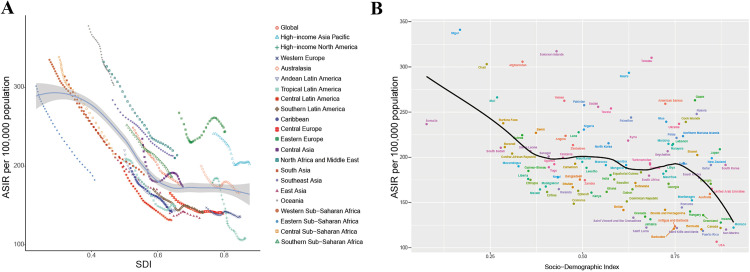
The correlation between the SDI index and age-standardized incidence rates for endometriosis in 21 GBD regions and 204 countries. **(A)** The correlation between the SDI index in 21 GBD regions and the age-standardized incidence rates for endometriosis; **(B)** The correlation between the SDI index in 204 countries and the age-standardized DALYs rates for endometriosis.

### Relationship between SDI and endometriosis burden

Fig 4 presents scatter plots of SDI values against age-standardized incidence rates, age-standardized prevalence rates, and age-standardized DALYs rates of endometriosis for 21 GBD regions and 204 countries. First, among the 21 GBD regions, regions with higher SDI values showed lower age-standardized incidence rates for endometriosis, suggesting that regions with relatively higher social development levels have a lower disease burden of endometriosis (Fig 4A). A similar pattern was observed in the scatter plot for 204 countries, where countries with higher SDI indices had lower age-standardized incidence rates of endometriosis, confirming the previous results (Fig 4B). Subsequently, the analysis of age-standardized prevalence rates and age-standardized DALYs rates for endometriosis revealed that as the SDI index increases, the disease burden of endometriosis gradually decreases (S2 Fig).

## Discussion

From 1990 to 2021, the incidence, prevalence, and DALYs rates of endometriosis showed an overall decline, suggesting a gradual reduction in disease burden. However, the impact still differs among reproductive-aged women of various ages. A non-linear relationship was observed between endometriosis burden and SDI levels, likely influenced by medical access, public health policies, cultural factors, and health awareness. Decomposition and frontier analyses were further applied to identify key factors and assess disease control across SDI regions.

Our study found that the incidence of endometriosis is higher in the age groups of 20−24 and 40−44. A cross-sectional observational study found that the proportion of endometriosis patients in the 21−24 age group was significantly higher than in those under 20 [25]. At present, a definitive diagnosis of endometriosis still requires laparoscopy combined with pathological confirmation. Patients under 20 years old often refuse transvaginal ultrasound due to sexual activity concerns, which leads to some early-stage endometriosis being missed [26,27]. Additionally, some endometriosis patients often visit the hospital due to symptoms of dyspareunia, which is one of the main reasons why women aged 20−24 are diagnosed with endometriosis [28]. Moreover, with age, women’s fertility gradually declines, which leads them to undergo more health check-ups and fertility assessments during this stage, thereby uncovering potential endometriosis issues [29]. Women aged 30–40 years show relatively lower incidence and prevalence of endometriosis. This may result from pregnancy, breastfeeding, and hormonal contraceptive use, which suppress menstruation and mask symptoms, leading to underdiagnosis. After age 40, discontinuation of contraceptives and infertility evaluations often reveal previously undetected disease, making symptoms more apparent.Collectively, these factors may provide a plausible explanation for the differences in diagnostic patterns across age groups observed in the present study [30,31]. The COVID-19 pandemic significantly disrupted gynecological healthcare services worldwide, directly affecting the detection, diagnosis, and treatment pathways for endometriosis. Studies have shown that in 2020, the median surgical volume per center declined by 51.0%; although this situation improved in 2021, it still remained 33% below pre-pandemic levels [32]. It is therefore reasonable to infer that, in the final years of our study period (2020–2021), pandemic-related service interruptions, surgical delays, and changes in healthcare-seeking behaviors may have led to underdiagnosis of endometriosis cases, thereby introducing short-term shifts in the observed trends for incidence, prevalence, and DALYs.

Endometriosis is also associated with the incidence of chronic cardiovascular and metabolic diseases [33,34], with higher risks of hypertension, coronary heart disease, arthritis, and type 2 diabetes [35–37]. Some studies have found association between endometriosis and an increased risk of type 2 diabetes, with the risk potentially varying based on the history of infertility [38]. Some scholars believe this phenomenon is attributed to the high-inflammatory environment present in the bodies of endometriosis patients [39]. As endometriosis progresses and complications arise, this may be one of the reasons for the slight increase in incidence in the 40–44 age group. Future clinical researches could explore the specific causes of this phenomenon. The occurrence of endometriosis-related tumors is associated with the low estrogen state in postmenopausal women [40]. During pregnancy, elevated levels of estrogen and progesterone often ameliorate the clinical symptoms of endometriosis; however, studies have also shown a close association with adverse obstetric complications [41–44].

In this study, we found that both the incidence and prevalence of endometriosis showed a marked decline in the 45–49-year age group. We speculate that this pattern may be related to the decrease in circulating estrogen levels among women approaching menopause, as estrogen-dependent ectopic endometrial lesions become less active in the absence of hormonal stimulation, leading to lower incidence and prevalence in this age group [45]. Notably, ectopic endometrial tissue, particularly ovarian endometriotic cysts, can accelerate ovarian aging and impair ovarian function through mechanisms such as oxidative stress, DNA damage, and fibrosis [46], thereby increasing the risk of early menopause and further elevating the risk of chronic diseases such as osteoporosis and cardiovascular disorders [47,48]. Decomposition analysis revealed regions where aging markedly influences endometriosis. In these areas, implementing targeted and individualized care with long-term follow-up for peri- and post-menopausal women, including symptom monitoring, endocrine and reproductive assessment, and management of bone and cardiovascular health, is essential to lessen the combined burden of aging and endometriosis.

Our study examined the endometriosis burden across different SDI levels and found it was mainly concentrated in low-SDI regions.Variations in healthcare resources, economic status, and cultural practices influence awareness and attention to endometriosis, thereby affecting both disease burden and patients’ quality of life [49]. Variations across regions in the availability of imaging and laparoscopy, referral pathways, and reimbursement policies directly influence the suspicion and diagnosis of endometriosis, thereby creating geographic differences in diagnostic delay and apparent prevalence [3,50]. In addition, socioeconomic factors such as income, education, and insurance coverage shape patients’ willingness to seek care and continuity of care, increasing the likelihood of symptomatic rather than etiologic evaluation and, in turn, diminishing public and clinical attention to the disease [51,52]. It is worth noting that due to differing cultural norms in various countries and regions, people’s perceptions and concepts of pain vary, which indirectly affects the diagnosis rate of endometriosis [53]. Decomposition analysis showed that population growth and aging were the main contributors to the endometriosis burden. Population factors had a stronger impact in low-SDI regions, indicating that demographic structures indirectly influence disease levels. Targeted gynecological healthcare should therefore be prioritized in areas most affected by these factors to reduce the burden [54]. Future efforts should focus on how population structure and social development influence the burden of endometriosis. Targeted policies and public health interventions can help reduce the health and socio-economic burden on reproductive-aged women, as endometriosis causes substantial medical costs and productivity losses. This study provides new insights for its prevention and control.

## Conclusion

Overall, endometriosis is one of the significant health threats to reproductive-aged women. From 1990 to 2021, the incidence and prevalence of endometriosis in reproductive-aged women showed a slight decreasing trend. There are differences in the disease burden of endometriosis between different countries and regions. It is imperative to improve awareness of endometriosis to raise early diagnosis rates, thereby decreasing its health burden on women of reproductive age and reducing fertility risks.

### Limitations

Based on the GBD 2021 database, this study was the first to analyze changes in the incidence, prevalence, and DALYs rates of endometriosis among women of reproductive age at the global, regional, and national levels; however, several limitations of this study warrant discussion. First, due to differences in diagnostic capacity, disease awareness, and healthcare resource allocation across countries and regions, mild or asymptomatic cases of endometriosis may be underrecognized and underreported, leading to a potential underestimation of the true prevalence in the GBD data. Second, the diverse sources of GBD data may to some extent affect the comparability and accuracy of the results. Third, given that the GBD data collection adheres strictly to ICD disease coding criteria, certain subtypes of endometriosis may not be adequately captured. This methodological constraint could contribute to discrepancies between the GBD estimates and the actual epidemiological burden observed in real-world clinical settings.

## Supporting information

S1 Fig The decomposition analysis of endometriosis globally, across 5 SDI regions, and 21 regions from 1990 to 2021; The black dots represent the overall change values due to population growth, aging, and epidemiological changes. (A) Prevalence rates globally and in 5 SDI regions; (B) Prevalence rates in 21 GBD regions; (C) DALYs rates globally and in 5 SDI regions; (D) DALYs rates in 21 GBD regions.
(PDF)

S2 Fig The correlation between the SDI index and endometriosis disease in 21 GBD regions and 204 countries. (A) The correlation between the SDI index in 21 GBD regions and the age-standardized prevalence rates for endometriosis; (B) The correlation between the SDI index in 21 GBD regions and the age-standardized DALYs rates for endometriosis; (C) The correlation between the SDI index in 204 countries and the age-standardized DALYs rates for endometriosis; (D) The correlation between the SDI index in 204 countries and the age-standardized prevalence rates for endometriosis.
(PDF)
