# Supplementary figures and images for: Global, regional, and national burden of endometriosis among women of reproductive age, 1990–2021: Insights from the global burden of disease study 2021
Source: PLoS One. 2025 Nov 26;20(11):e0337074. doi: 10.1371/journal.pone.0337074 (PMC12654899; doi:10.1371/journal.pone.0337074)

A

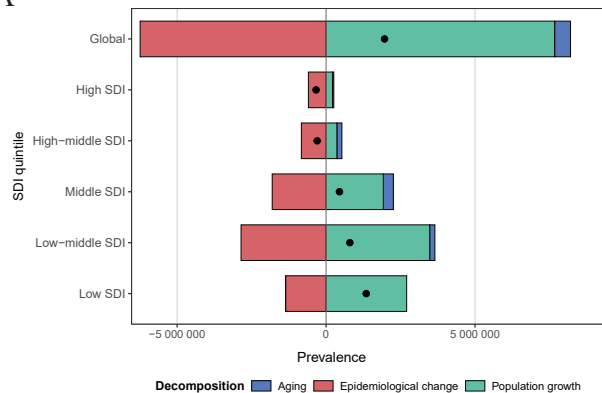

B

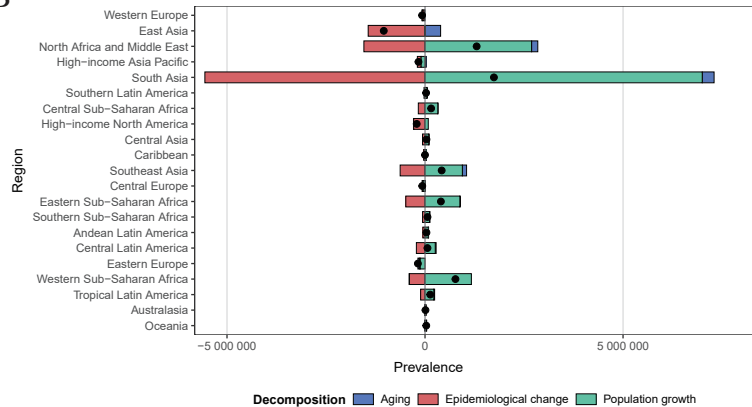

C

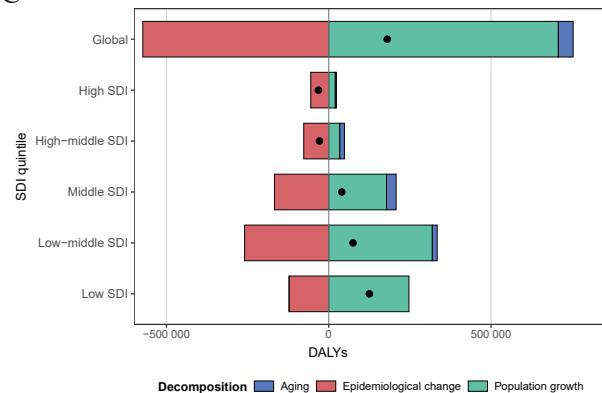

D

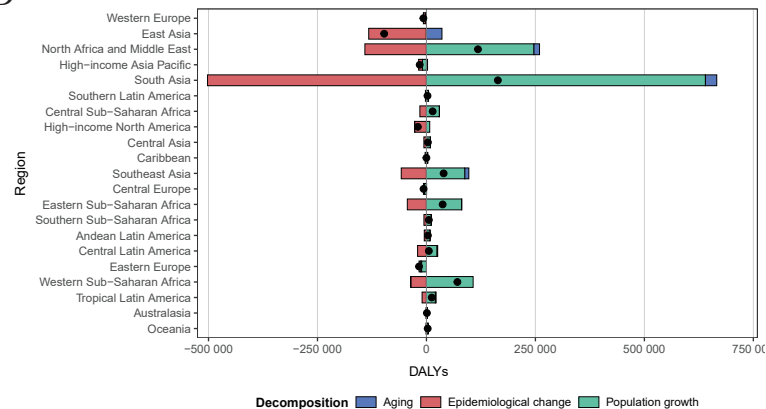

Supplement: S1 Fig — (A) Prevalence rates globally and in 5 SDI regions; (B) Prevalence rates in 21 GBD regions; (C) DALYs rates globally and in 5 SDI regions; (D) DALYs rates in 21 GBD regions. (PDF) [file pone.0337074.s001.pdf]

B

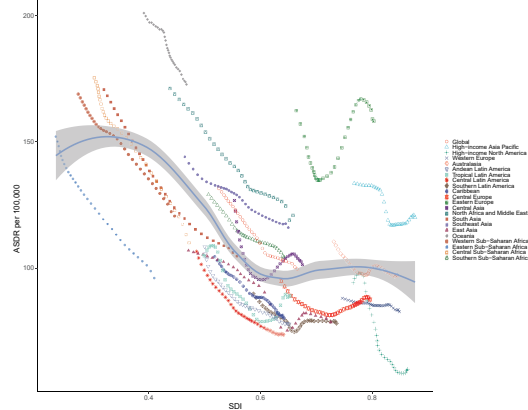

D

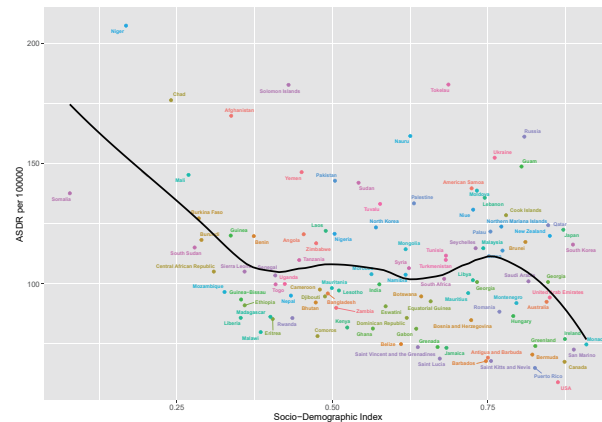

Supplement: S2 Fig — (A) The correlation between the SDI index in 21 GBD regions and the age-standardized prevalence rates for endometriosis; (B) The correlation between the SDI index in 21 GBD regions and the age-standardized DALYs rates for endometriosis; (C) The correlation between the SDI index in 204 countries and the age-standardized DALYs rates for endometriosis; (D) The correlation between the SDI index in 204 countries and the age-standardized prevalence rates for endometriosis. (PDF) [file pone.0337074.s002.pdf]
